# Self-Reported Engagement in a Drug Prevention Program: Individual and Classroom Effects on Proximal and Behavioral Outcomes

**DOI:** 10.1007/s10935-018-00532-1

**Published:** 2019-01-10

**Authors:** William B. Hansen, Charles B. Fleming, Lawrence M. Scheier

**Affiliations:** 1 Prevention Strategies, LLC, 5900 Summit Ave., Browns Summit, NC 27214 USA; 2 Social Development Research Group, University of Washington, 9725 Third Ave., NE, Suite 401, Seattle, WA 98115 USA; 3 LARS Research Institute, Inc., 15029 N Thompson Peak Pkwy B111, Scottsdale, AZ 85260 USA

**Keywords:** Student engagement, Prevention, Alcohol, Drunkenness, Cigarettes, Classroom size

## Abstract

Numerous studies emphasize the role of student engagement in academic learning and performance. Less known is whether engagement plays a role in drug prevention program outcomes. We examined a self-report measure of engagement as part of the *All Stars Core* drug prevention program evaluation, assessing its impact on target risk mechanisms and behavioral outcomes. Students completed pretests just prior to and posttests just after completing the intervention. Surveys assessed demographics, proximal intervening measures (i.e., commitments to avoid substance use and antisocial behavior, perceived lifestyle incongruence with substance use and antisocial behavior, normative beliefs about substance use and antisocial behavior, and parental attentiveness), and distal outcome measures of alcohol, cigarette use, and antisocial behaviors. A brief 6-item posttest measure including items tapping the students’ perspective on the quality of teaching the program material and their level of engagement with the program was internally consistent (α = .79). Multi-level analyses positing engagement effects at both the classroom- and individual-level indicated that classroom average engagement was significantly associated with all the targeted risk mechanisms, and outcomes of antisocial behavior and alcohol use, controlling for pretest measures and classroom size. Individual student engagement relative to classroom peers was significantly associated with all posttest target risk mechanisms and behavioral outcomes. The current findings suggest that students should routinely provide assessments of engagement and perceived quality of teaching, which would improve our understanding of how prevention programs work. Teachers can improve engagement by paying attention to students when they speak in class, making the program enjoyable to participants, encouraging students to share opinions, stimulating attentiveness, being well prepared to deliver the intervention, and helping students think broadly about implications of drug prevention as it affects their lives. This type of support will ultimately engage students in ways that will enhance the likelihood that these programs will have their desired effects.

## Introduction

School-based drug prevention programs are increasingly being disseminated, most notably as they achieve the desired evidence-based benchmark. Notwithstanding, this benchmark does not guarantee effectiveness when the programs are adopted for local implementation. Indeed, the literature is replete with documentation that quality of delivery and fidelity of implementation are crucial to achieving program goals (Durlak & DuPre, 2008). Among the implementation factors proposed to account for program effectiveness is student engagement. To date, there has been only limited examination of student engagement as a potential moderator of program effectiveness. The goal of this paper is to examine engagement using extant data collected from students who received a disseminated program, *All Stars Core* (Hansen, 2015). We examine the role of self-reported engagement in accounting for proximal outcomes targeted by the program and also focal behaviors. Because *All Stars Core* is delivered in classroom settings, we examine both individual-level and classroom-level models of engagement.

School-based drug prevention has amassed over 40 years of program evaluation findings suggesting that a wide range of programs work to reduce youthful drug use (Scheier, 2015). This body of evidence is inclusive of different program modalities and has been subject to extensive meta-analysis (e.g., Gottfredson & Wilson, 2003; Tobler et al., 2000). As diffusion of innovations occurs and the next generation of programs becomes more commonplace, the focus shifts from efficacy evaluations toward examining “for whom and under what conditions” these programs work most effectively (Botvin & Griffin, 2010; Gottfredson & Wilson, 2003). This is part of a continued interest emphasizing implementation factors that influence program success. Research shows, for instance, that implementation success may vary in response to school climate (Beets et al., 2008) and organizational support (Kam, Greenberg, & Walls, 2003), teacher adaptations of program curriculum (Hansen et al., 2013; Miller-Day et al., 2013), participant responsiveness (Stephens et al., 2008) or some combination thereof to account for program outcomes. These emphases also comport with the field’s need to understand why some programs have only modest effects when delivered in real-world settings (Miller-Day et al., 2013; Ringwalt et al., 2013).

During classroom time, drug prevention programs rely heavily on student interactions to reinforce newly acquired skills. For instance, students actively engage in role-play skits where they can practice social assertiveness and drug refusal skills. Often there are group problem-solving tasks that boost competence, homework assignments that involve parental support, and some form of classroom interaction and cooperative learning where students challenge drug use norms, discuss expectations, engage in values clarification, and learn about the negative sequelae of drug use. In many cases, students receive constructive feedback from teachers and their peers, an instructional strategy reflecting the tenets of social learning theory (Bandura, 1986). These efforts require that students enjoy participating in the program, find the program materials attractive, and willingly participate through active discussion where they can ask questions and learn. Students who are psychologically invested in the program are more likely to learn the new material and master the skills that are fundamental to drug prevention (e.g., social skills that promote drug refusal efficacy).

In order to better understand the role of student engagement in drug prevention, we first briefly examine how student engagement is conceptualized in the education literature. This includes examining the role of engagement in academic outcomes and high-risk behaviors. We then briefly examine different strategies for assessing student engagement and how they have been utilized in studies of drug prevention. We then present findings from a school-based drug prevention program conducted in Northern Ireland and that assessed student-level measures of engagement in relation to both proximal (intervening variables) and distal (behavioral) outcomes.

## Conceptual Models of Student Engagement

Student engagement is a popular concept in education, as it bears heavily on student achievement and because of its implications for classroom instruction (Appleton, Christenson, & Furlong, 2008; Fredericks, Blumenfeld, & Paris, 2004). Historically, interest in student engagement was born out of concern in the lackluster performance of students and declines in achievement scores (Newmann, 1992). In response to critiques of the American education system (Bloom, 1987), educators became interested in what separated students who were engaged in their classroom educational experience from those that were disengaged, bored, cutting class, and disrupting their peers (Appleton et al., 2008; Griffiths, Lilles, Furlong, & Sidhwa, 2012). Disengaged students have much higher rates of school dropout (Archambault, Janosz, Morizot, & Pagani, 2009) and substance use (Bond et al., 2007), and experience other negative developmental outcomes (Archambault, Janosz, Fallu, & Pagani, 2009; Carter, McGee, Taylor, & Williams, 2007). Furthermore, the educational literature also shows that students who do not have a committed and caring adult become disaffected from academic pursuits leading to absenteeism, lackluster performance in terms of coursework, and eventual participation in unconventional behavior including drug use and school dropout (Archambault, Janosz, Fallu, et al., 2009; Archambault, Janosz, Morizot, et al., 2009; Fredericks et al., 2004). Conversely, when students perceive their teachers as supportive and respectful (what has been termed ‘pedagogical caring’) this tends to increase engagement (Reddy, Rhodes, & Mulhall, 2003).

No one perspective dominates the student engagement literature. Some view it as an internal state reflecting essential characteristics of the individual, their sense of autonomy, control, and relatedness (Connell & Wellborn, 1991). Others suggest that student engagement reflects institutional practices (the school environment) and teacher influences in addition to internal motivations. Regardless of orientation, many consider engagement to reflect “psychological processes” capturing the “attention, interest, investment, and efforts students expend in the work of learning” (Marks, 2000, p. 154). Students who pull their weight, are personally committed to schooling, and do well in class, may be more highly motivated and earnest in pursuing their academic goals. They place greater effort into their learning tasks, have more positive emotions, and show greater interest in learning (Patrick, Ryan, & Kaplan, 2007). This holds true for drug prevention, where students who enjoy the teaching methods and program content may benefit from the classroom instruction compared to students who are less engaged. In other words, students who are engaged are more likely to identify with the program goals, understand the instructional value of the program, and participate in activities that can promote self-growth and learning. This leads to a conceptual model that posits that highly engaged students who enjoy the program content will fare better in terms of program outcomes.

The concept of student engagement has also found a foothold in drug prevention. For instance, Berkel, Mauricio, Schoenfelder, and Sandler (2011) proposed an integrated model of implementation that suggests that participant responsiveness is one of several factors that bear on program success. Participant responsiveness (known in the model as *engagement*) is generally reflected by four possible indicators: attendance, active participation, home practice, and satisfaction. In an educational framework, participation references the day-to-day behaviors that reflect students’ role in school including their levels of interest in the curriculum. For purposes of this paper, and consistent with research on engagement in academic settings (Fredricks & McColskey, 2012; Jimerson, Campos, & Greif, 2003), engagement is conceptualized as student response-centered and includes their attentiveness, satisfaction, enjoyment and perceived meaningfulness of the program, and their recall of teacher interactions and the teacher’s enthusiasm or skill in teaching the program.

### Engagement and Drug Prevention Program Effectiveness

Research shows that among the cornerstones of achieving high fidelity is delivering an intervention by stringently adhering to the lesson activities and also teaching in a manner that is highly engaging and evokes student participation (Dane & Schneider, 1998; Dusenbury, Brannigan, Falco, & Hansen, 2003). Rather than having students be passive recipients of drug prevention, meta-analyses consistently document that highly interactive programs net greater success than do programs using didactic teaching strategies (Tobler, 1986; Tobler et al., 2000). Several recent studies broaden the research base with regard to the role of engagement and factors that promote engagement. For example, studies reinforce that authoritative teachers^1^ (those who are attentive and warm and who set high standards for participation and performance) are likely to have increased engagement from their students (Edmunds, Ntoumanis, & Duda, 2008; Walker, 2008). Teachers invariably bring a certain disposition to the classroom and their teaching style can work to motivate students to achieve at higher levels. Certainly, in terms of drug prevention, carryover from their classroom management strategies can serve as impetus for students to engage drug prevention materials. However, teachers represent only one aspect of the equation and there may be structural factors at play in promoting student engagement. For instance, classroom size may influence student engagement (Blatchford, Bassett, & Brown, 2011; Dee & West, 2011; Nye, Hedges, & Konstantopoulos, 1999), with smaller classrooms usually considered superior because they are conducive to more frequent social interactions. However, most of the research in this area has emphasized comparisons between very large classrooms (e.g., 30 or more students) and considerably smaller classrooms.

### Assessing Student Engagement in Drug Prevention

Studies that have attempted to assess student engagement rely either on teachers’ self-ratings, observer ratings using classroom video recordings, or the students’ self-reports (Dusenbury et al., 2003). While each strategy has strengths and weaknesses, they share the common goals of assessing the quality of intervention delivery, whether the teacher used appropriate instructional strategies, if the teacher was enthusiastic, and if the teacher was engaged with students. Teachers’ self-report ratings of engagement (Bishop et al., 2014; Low, Van Ryzin, Brown, Smith, & Haggerty, 2014) have long been suspect as providing a biased accounting of performance (Hansen & McNeal, 1999; Hansen, Pankratz, & Bishop, 2014; Miller-Day et al., 2013). In fact, Lillehoj, Griffin, and Spoth (2004) showed there is little concordance between teacher ratings and trained observer ratings of fidelity, the latter of which more efficiently predicted drug use outcomes. Despite the incongruities, teachers’ self-reports of student engagement have been used with some success to predict program outcomes. For instance, Low et al. (2014) reported that teachers’ ratings of student engagement (i.e., student was engaged, absorbed material, and was easy to manage) was significantly related to the intermediate outcomes in a bullying prevention program.

Observational methods provide an independent evaluation source, thus reducing social desirability bias from teachers’ ratings of their own performance. However, observational techniques that use small segments of video recordings to monitor teacher engagement levels capture only a slice of the classroom experience, leaving coders faced with the challenge of assessing the parts among the whole (e.g., only the teacher’s behavior, not the student’s; Hansen & McNeal, 1999). In most cases, video recordings based on a sampling of whole sessions or specific activities are used to gauge the teacher’s enthusiasm and how often they use certain interactive student-centered techniques (Giles et al., 2008; Gottfredson, Cross, Wilson, Rorie, & Connell, 2010; Pettigrew et al., 2013), but does not capture the students’ responsiveness or attentiveness. Giles et al. (2008) reported that more expressive teachers who asked students more questions and used more motivational techniques netted superior program outcomes. Bishop et al. (2014) used observational methods to make summary judgments about levels of student engagement and found very low inter-rater agreement, suggesting that observational methods may not always be ideal for assessing student engagement in prevention programs.

In contrast, Pettigrew et al. (2013) reported high reliability of student engagement measures using observational video recording methods. Ratings classified students as disconnected, attentive or participatory, and teachers as being passive (lack of control and exhibiting a poor management style), strict (control at all times) or coordinated (general control while allowing students autonomy to complete activities). Utilizing the same intervention data, Shin and her colleagues (Shin, Miller-Day, Pettigrew, Hecht, & Krieger, 2014) focused exclusively on ratings of the teachers’ engagement efforts by using video recordings to assess their attentiveness to students, energy level, and whether the teacher was expressively positive during lesson delivery. The authors conclude that quality of program delivery involves the degree of engagement for both teacher and student. More recently, Pettigrew et al. (2015) expanded their video observational coding scheme using the same *keepin’ it REAL* drug prevention program to include reliable assessments of both teacher engagement (attentiveness, enthusiasm, seriousness, clarity, and positivity) and student engagement (attention and participation), reinforcing that higher engagement at both levels produced more optimal prevention outcomes.

Ratings obtained from students, which we utilize in the current study, not only give a direct sense of the students’ impression of how the program was delivered, but also introduce some bias because of the past instructional history between the student and teacher. Highly engaged students are likely to receive greater attention from their teachers, including positive feedback, whereas disaffected students may not experience the same levels of encouragement or support (Skinner & Belmont, 1993). Nevertheless, this technique has been used to show, for instance, that student ratings of program enjoyment, attentiveness, and quality of student–teacher relationship were significantly and positively associated with proximal outcomes for a drug prevention program (Giles, Harrington, & Fearnow-Kenney, 2001). In a study examining the impact of teacher communication styles on program outcomes, Giles et al. (2012) found that students of teachers who rated themselves as “authoritarian” reported less program engagement and poor student–teacher relationships, whereas students of teachers who rated themselves as “expressive” reported more immediacy, program engagement, and better student–teacher relationships. Other fidelity of implementation studies have utilized student ratings of “*program acceptance*” and “*quality of the implementer*” as proxies for student engagement in their process evaluation; however, they have either failed to link these student ratings with either intermediate or distal outcomes (Lisha et al., 2012) or have reported non-significant relations (Rohrbach, Dent, Skara, Sun, & Sussman, 2007).

### Importance of the Current Study

We believe that conceptual models of student engagement relevant to academic settings as well as the different approaches to assessing student engagement have potential application to drug prevention programs. Bishop et al. (2014) recently reported that, of all the characteristics of implementation assessed in an observational study (dosage, adherence, quality of program delivery, adaptation, and participant engagement), assessing engagement had the lowest inter-rater agreement. This underscores the need for psychometrically reliable tools for assessing engagement. This is particularly true when onsite observers are not available, as is the case with most disseminated drug prevention programs. Our study addresses these concerns, first by examining the psychometric properties of a student self-report measure of engagement. We then empirically examine whether students’ cognitive appraisal of the program, comprised of their own self-reported attentiveness and interaction and their regard for the teacher’s investment in the program, contributes uniquely to program outcomes. Furthermore, we examine effects of engagement on both proximal and distal program outcomes at both the individual and the classroom levels.

Whole classrooms may vary in the extent to which they are responsive to intervention content due to variation in teachers’ presentation skills, class composition, or dynamics, and the influence students within a classroom have on one another. Classrooms can also vary in their sense of belonging or identification with the goals of learning (Goodenow, 1993; Ryan & Patrick, 2001), as demonstrated by studies of classroom contextual effects on motivation or engagement (Patrick et al., 2007; Urdan & Schoenfelder, 2006). Drug prevention programs do not just influence the individual student but also seek to institute social-contextual changes that involve the whole classroom. This is particularly true if normative education is a featured targeted proximal variable. That is, interventions that correct misperceptions about substance use prevalence and acceptability intend to change individual as well as classroom-level normative beliefs.

Using these strategies effectively, classrooms characterized as cooperative and socially cohesive, and that provide a supportive learning environment, may have better outcomes because students work together toward common learning goals (Slavin, Hurley, & Chamberlain, 2003). This may not be true for students who attend classrooms that are disruptive, lacking formal structure, and lack oversight. In the current study, we examine the role of student engagement using a multi-level framework, allowing us to estimate effects coinciding with the nesting of students within classrooms (Reyes, Brackett, Rivers, White, & Salovey, 2012). This provides a means to partition variation in engagement into between-classroom and -student variation. We assess the unique effects of engagement at each level on both proximal intervention targets (the hypothesized active ingredients of *All Stars Core*) and the focal behavioral outcomes of substance use and antisocial behavior. The latter dual focus arises because drug use and antisocial behavior tend to co-occur (Jessor & Jessor, 1977) and may share etiological pathways, with student engagement factoring into both outcomes. We also control for classroom size in order to understand its possible complicit role in engagement and as a potential rival alternative explanation of program outcomes. Overall, this work should assist with identifying strategies that teachers may adopt to improve engagement among their students.

## Method

### Subjects and Setting

Students were participants in a school-based drug prevention project funded by the U.K. Big Lottery and implemented and evaluated independently by Barnardo’s Northern Ireland, an action oriented non-governmental philanthropic organization in the U.K. Students in 62 classrooms receiving *All Stars Core* (Hansen, 2015) completed surveys. Data were available from 980 students, 975 of whom (99.5%) provided both pretest and posttest surveys. Slightly more than half were males (57.9%). Students were 11 (44.9%), 12 (48.9%), or 13 (6.2%) years old at pretest.

Because the project originated in Northern Ireland, program administrators collected data on community background. About half (50.7%) of students were from the Protestant community, 39.1% were from the Catholic community, and 10.3% said they were from neither one.

#### *All Stars Core*

Briefly, *All Stars Core* is a program consisting of 13 45-min lessons ideally delivered to 11-, 12-, or 13-year-old students. *All Stars Core* lesson content consists of three broad program components, each of which targets measures that represent the active ingredients of the program (intermediate outcomes). Changes in these proximal outcomes are hypothesized to lead to reductions in drug use (Hansen, 2015; McNeal, Hansen, Harrington, & Giles, 2004). The three principal active ingredients of the program include: (1) lifestyle incongruence with risky behaviors; (2) normative beliefs about alcohol, cigarette and marijuana use and bullying; and (3) intentionality, or commitments to avoid substance use and violence. Each of the targeted proximal outcomes are tied to instructional strategies represented by a series of in-class activities. In addition to classroom lessons, out-of-class take home assignments address a fourth intermediate outcome (4) parental attentiveness through parent–child communication. For an intervention to be effective, it is crucial that targeted proximal variables have a strong relationship with behaviors of interest and that the program is capable of having a sizable impact on these variables (Hansen & McNeal, 1996). The intervention addresses developing plans for a positive future; building a perception that alcohol, tobacco and other drug use and violent activities would interfere with that future; helping clarify that substance use is uncommon and thought of as unacceptable among the peer group; and encouraging students to make voluntary commitments to avoid risky behaviors. Intervention strategies that make up the action theory component include role-plays, class discussions, games, and small group activities (e.g., debates). The program includes minimal didactic lectures. Homework assignments ask students to review class materials, engage in discussion with parents or other respected adults using prompts provided on worksheets, and return written comments to the teacher. These homework assignments provide parents and other important adults the opportunity to forge bonds and communicate about relevant issues discussed in class. The program used was adapted from the U.S. version for use in the U.K. to match linguistic and cultural norms. Thirty-seven teachers from 14 schools delivered the program. Schedules for implementation varied, but typically involved delivery of the entire program’s 13 sessions once a week during either the fall or spring term.

#### Measures and Scale Creation

In addition to demographic items (gender, age and community identification: Catholic or Protestant), the survey included three indicators of *Substance Use*, dichotomous items that assessed past 30-days alcohol use, drunkenness, and cigarette smoking. Responses were coded “0” for non-behavior and “1” for reporting behavioral activity. Students also provided self-reports regarding whether they engaged in antisocial behaviors (being angry, fighting, lying, and cheating) with a response format of “not true,” “sort of true,” and “certainly true,” coded as 0, 5 and 10, respectively. Being angry, fighting, lying, and cheating scores were averaged to form an *Antisocial Behavior* scale with possible values ranging from 0 (not antisocial) to 10 (highly antisocial).

Measures of the target risk mechanisms were based on scale scores ranging from 0 to 10, with higher scores being theoretically more desirable. A copy of all targeted proximal variable survey items are listed in the Appendix. *Commitment to Avoid Drug Use* score was formed from eight items (α = .77) that assessed a student’s intentions regarding future alcohol and cigarette use (e.g., “I would be willing to sign my name to a pledge saying that I will not use alcohol”). A *Commitment to Avoid Fighting* score was formed from four items (α = .70) that assessed intentionality regarding physical fighting (e.g., “I am committed to solving disagreements peacefully”). A *Lifestyle Incongruence With Drug Use* score was formed from six items (α = .67) that assessed student’s beliefs that alcohol, cigarettes, and other drug use would interfere with lifestyle goals and preferences. One item on this scale is “Using marijuana would keep me from accomplishing my goals.” The *Lifestyle Incongruence With Fighting* score was formed from two items (α = .53) that assessed the degree to which fighting would fit with their desired lifestyles (e.g., “Fighting does not fit with the life I want to live”). *Normative Beliefs About Drug Use* was formed from seven items (α = .75) that assessed perceived prevalence among their peer group of alcohol, cigarette, and other drug use (e.g., “How many people your age do you think get drunk at least once a month?”) and the acceptability of substance use to the student’s friends (e.g., “My friends think smoking marijuana is a stupid thing to do”). *Normative Beliefs About Fighting* was formed from 4 items (α = .65) that assessed prevalence (e.g., “How many people in your school get into fights?”) and acceptability of fighting (e.g., “My friends think it is not okay to fight to solve problems”). *Parental Attentiveness* was formed from seven items (α = .79) that assessed parental monitoring (e.g., “My parents always know who I’m with”) and parental communication about risky behaviors (e.g., “I have talked with my parents about living a drug- free life”). A *Composite Mediator Score* was created by averaging values from all sub-scales (α = .85). Survey items that referred to substance use were modified from a standardized U.S. national survey. Items that addressed behaviors and attitudes about violence were newly created for use in the U.K.

In addition, at posttest, a six-item *Student Engagement* scale assessed students’ perception of the teacher’s enthusiasm (two items), and single items assessing enjoyment, student in-class activity, attentiveness, and program value. Engagement items and response categories and weights are presented in the Appendix. All items loaded on a single factor (eigenvalue = 2.95) and the estimate of internal consistency was α = .79. Summary scores for the *Student Engagement* scale thus had possible values from 0 to 10 with higher values reflecting greater engagement.

#### Survey Administration

Students completed pretest surveys within a week prior to receiving the program. They completed posttest surveys shortly after the conclusion of program delivery. Barnardo’s staff, who were not directly involved in teaching the program, administered the surveys. Instructions on the cover sheet of the survey assured students their responses would be confidential. Barnardos owns the data and shared them with the research team for the purposes of conducting these analyses. Missing data from a failure to complete items on both pretest and posttest surveys was quite minimal, ranging from 5 of 980 (0.51%) to 20 of 980 (2.05%).

#### Analysis Plan

We used SPSS 20.0 (2011) to estimate reliability coefficients and zero-order correlations between pretest and posttest scores on the target risk mechanisms. We used HLM 6.0 (2009) to test multilevel models that partitioned between- and within-classroom variance (Raudenbush & Bryk, 2002). Descriptive analyses used intercept-only models with no predictors. A multilevel linear model regressed engagement on classroom size and classroom size squared, the latter to capture any curvilinear effects. Multilevel linear regression modeled the prediction of continuous posttest intervening measure scores and antisocial behavior, while multilevel logistic regression models repeated this test assessing unique predictors of posttest substance use. In these models, we included classroom average engagement score and classroom size as classroom-level measures predicting classroom variation in intercepts, while student engagement was a group-centered individual-level predictor, along with pretest score on the given outcome. We treated both individual-level engagement and pretest score (for the relevant outcome) as fixed effects. To aide in the interpretability of the conditional models, we standardized all continuous variables. We standardized the classroom-level variables with respect to between-classroom variation. We also standardized outcomes and individual-level variables with respect to total variation. Where appropriate, we calculated intraclass correlations (*ICC*) as an indicator of the magnitude of clustering given the hierarchical structure of the data.

#### Protection of Human Subjects

Barnardo’s procedures adhered to U.K. standards for human subjects’ protection. Pretest and posttest surveys included matching ID numbers. Barnardo’s survey administrators used a printed roster that listed ID numbers and provided a place to write names. Survey administrators used these rosters only to ensure that individuals completed pretest and posttest surveys with matching ID numbers. Survey administrators did not forward class rosters to the research team and rosters were destroyed after posttest data collection. As a consequence, the data provided to the research team was de-identified. Likewise, researchers received no classroom identifying information. Because this research involved the secondary analysis of archived de-identified data, this study was determined to be exempt from the need for oversight based on 45 CFR 46.101(b) 4; the chair of the Tanglewood Research IRB confirmed this exempt claim.

## Results

### Prevalence of Behaviors

Substance use was low among classrooms. At pretest, only 6.7% of students reported drinking alcohol in the past 30 days, 1.9% reported getting drunk, and 1.3% reported smoking cigarettes. At posttest, 6.1% of students reported drinking alcohol in the past 30 days, 1.7% reported getting drunk, and 1.9% reported smoking cigarettes. At the classroom level, 43.5% of classrooms at pretest and 45.2% at posttest had no drinkers, 74.2% at pretest and 75.8% at posttest had no drunkenness, and 82.3% at pretest and 75.8% at posttest had no smokers. Unconditional multilevel logistic regression models indicated that variance between classrooms was statistically significant at the *p* < .05 level only for alcohol use at posttest.

The average classroom score on the *Antisocial Behavior* scale at pretest was 2.91 (between-classroom *SD* = 0.44, within-classroom *SD* = 2.36, *ICC* = .03), which decreased to 2.58 at posttest (between-classroom *SD* = 0.41, within-classroom *SD* = 2.26, *ICC* = .03). Between-classroom variance in antisocial behavior was significantly different from zero at both time points at the *p* < .01 level.

#### Proximal Outcomes

At pretest and posttest, there was significant variability between classes in students’ reports related to the program’s intervening measures (see Table 1), with variance terms ranging from 7% to 18% at the between-classroom level. For all of the proximal outcomes at both time points, between-classroom variance was significant at the *p* < .01 level. Significant differences suggest that the null hypothesis (all of the classrooms had the same mean scores) should be rejected.

**Table 1:** Pretest and posttest descriptive statistics

	Pretest				Posttest			
	Mean	Between *SD*	Within *SD*	ICC	Mean	Between *SD*	Within *SD*	ICC
Commitment to Avoid Drug Use	8.33	0.44*	1.53	0.08	8.28	0.44*	1.50	0.08
Commitment to Avoid Fighting	6.88	0.82*	2.10	0.13	7.10	0.79*	1.95	0.14
Lifestyle Incongruence With Drug Use	8.41	0.46*	1.61	0.08	8.42	0.43*	1.61	0.07
Lifestyle Incongruence With Fighting	7.99	0.73*	1.99	0.12	8.18	0.60*	1.86	0.09
Normative Beliefs About Drug Use	7.82	0.57*	1.45	0.13	7.67	0.65*	1.41	0.18
Normative Beliefs About Fighting	6.11	0.74*	1.92	0.13	5.99	0.78*	1.87	0.15
Parental Attentiveness	7.75	0.54*	1.70	0.09	7.77	0.51*	1.64	0.09
Composite Mediator Score	7.60	0.55*	1.24	0.16	7.62	0.55*	1.25	0.16

Between = between classroom, Within = within classroom, *SD* = standard deviation, ICC = Intraclass correlation. Estimates are from multilevel intercept-only models with variance partitioned into individual and classroom levels. Between-classroom variance is significantly greater than zero at the *p* < .01 level for all variables at each time point

There was also considerable pretest–posttest concordance among students’ scores as evidenced by the strong pretest–posttest correlations (see Table 2; all correlations were significant beyond *p* < .01). The strength of this across-time relationship diminished somewhat for *Lifestyle Incongruence With Fighting* compared to the other target risk mechanisms. Interestingly, the correlations between pretest intervening measures and the magnitude of pretest–posttest changes for classrooms were negative. This suggests that students with initially lower scores improved the most with respect to intervening measures (active ingredients) and that students with relatively higher scores generally remained the same. For example, while the pretest–posttest correlation for the *Composite Intervening Measure Score* was .54, the pretest-change-score correlation was − .48. Two statistical artefactual issues may come into play here, one being a ceiling effect preventing students with relatively high pretest scores from dramatically increasing their scores over time, and the second one concerning regression to the mean.

**Table 2:** Correlations between pretest mediator scores and posttest scores and pretest–posttest change scores

	Posttest	Pretest–posttest change
Commitment to Avoid Drug Use	.45	− .53
Commitment to Avoid Fighting	.45	− .57
Lifestyle Incongruence With Drug Use	.33	− .58
Lifestyle Incongruence With Fighting	.32	− .62
Normative Beliefs About Drug Use	.39	− .56
Normative Beliefs About Fighting	.44	− .54
Parental Attentiveness	.49	− .53
Composite Mediator Score	.54	− .48

Correlations are based on individual-level analyses

All correlations are significant at the *p* < .01 level

#### Classroom Size and Engagement

As noted in the introduction, several researchers have considered classroom size to be a potential factor affecting engagement and is thus a plausible rival hypothesis to consider. Students in very large classes (> 30 students) may find engagement problematic because they don’t experience as much teacher–student contact during class. We therefore examined the relationship between engagement and classroom size in a multilevel framework. An unconditional model showed engagement varied at both the between- and within-classroom level (mean = 7.51, between-classroom *SD* = 1.01, within-classroom *SD* = 1.87, *ICC* = .23), with the between-classroom variation significant at *p *< .01. Classroom size and classroom size squared (designed to capture any curvilinear effect) were entered as classroom-level predictors, and both were significant (classroom size coefficient = 0.46, *SE* = 0.16, *p* = .005; classroom size squared coefficient = − 0.12, *SE* = .004, *p* = .007). Across the range of classroom sizes in the study (minimum = 3, maximum = 27, average = 15.81), the trend was for slightly larger classrooms to fare better in terms of engaging students, but the marginal effect of additional students diminished as classes got larger; however, because classroom size accounted for relatively little variance, it was discounted as a primary predictor of student engagement.

#### The Effect of Engagement on Proximal Outcomes

We examined the influence of student engagement on posttest proximal outcomes that are the active ingredients of *All Stars Core*. These models were covariate adjusted for pretest risk factor scores and classroom size. Table 3 summarizes the results of these analyses. Because we used standardized measures for these models, the effect coefficients can be interpreted as the standard deviation unit change in the target risk factor associated with a one standard deviation unit change in the given independent variable. As expected, classroom size played a non-significant role as a predictor of posttest risk factor scores. On the other hand, even after accounting for pretest risk factor scores, student engagement at both the between- and within-classroom level accounted for significant variation in the classrooms’ posttest risk factor scores. This means that students attending the more engaged classrooms and students who reported more engagement than their classroom peers had higher scores on the posttest proximal outcomes, controlling for their pretest scores. These models accounted for between 55% and 85% of the between-classroom variance in target risk factors and 17% and 38% of the within-classroom variance.

**Table 3:** Results of multilevel linear regression estimating the influence of key indicators on posttest mediator scores

	Pretest	Engagement		Class Size	Variance explained	
		Classroom	Individual		Classroom	Individual
	Coef.	Coef.	Coef.	Coef.	%	%
Commitment to Avoid Drug Use	.36**	.19**	.34**	.03	76	27
Commitment to Avoid Fighting	.34**	.23**	.31**	.04	73	24
Lifestyle Incongruence With Drug Use	.27**	.17**	.33**	.03	68	17
Lifestyle Incongruence With Fighting	.24**	.22**	.37**	.00	67	18
Normative Beliefs About Drug Use	.31**	.27**	.24**	− .02	55	18
Normative Beliefs About Fighting	.35**	.27**	.26**	.01	78	21
Parental Attentiveness	.39**	.21**	.36**	.03	85	32
Composite Mediator	.39**	.27**	.40**	.02	80	38

*Coef.* = HLM generated coefficient. All variables were standardized. Coefficients represent the standard deviation unit change in the dependent variable associated with a one standard deviation unit change in the independent variable. Classroom-level engagement and class size were standardized with respect to between-classroom variation. Variance explained was calculated by comparing the variance estimates for unconditional intercept-only models with estimates for models including pretest score, engagement at individual and classroom levels, and class size as predictors

**p < .01

**Fig. 1:**
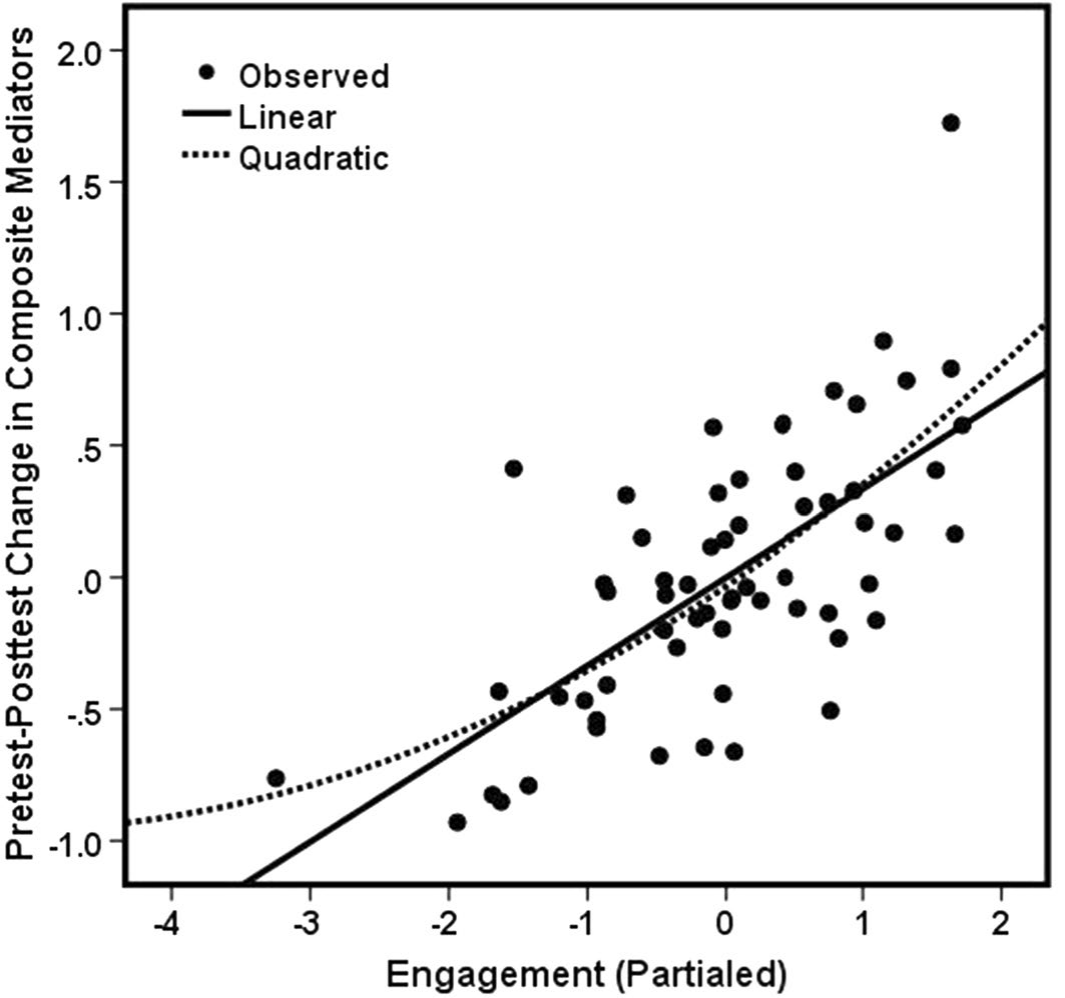
Relationship between engagement at the classroom level and pretest–posttest changes in composite mediator score with pretest scores partialed out

Figure 1 illustrates the effect of average classroom engagement (aggregated to the classroom level) on the composite risk factor scores (aggregated for pretest and posttest to the classroom level). The plot shows relationships between levels of classroom engagement and the residual variance in composite proximal outcome scores after accounting for pretest scores. Clearly, there is a distinct relation between student engagement levels, and how much the class, as a whole, improves on the targeted risk factors. Greater engagement is associated with more positive program results after controlling for pretest status and classroom size. Prior research has noted that each of the targeted proximal variables worsen as youth grow older (Hansen & Hansen, 2016). The goal of intervention is to reverse this trend. As Fig. 1 attests, this can occur when a program is delivered in an engaging manner but is likely to have no effect when engagement is poor.

#### Associations Between Engagement and Behavior

Table 4 shows that there was inconsistent support for significant relations between classroom average engagement and substance use behaviors. Being in a more engaged class was associated with being less likely to drink alcohol at posttest. Not surprisingly, because there was little between-classroom variation in other types of substance use, classroom average engagement was not a significant predictor of drunkenness or tobacco use. Engagement as an individual-level predictor was associated significantly with all measures of substance use, with more engaged students being less likely to report substance use at posttest (all *OR*s < 1.0).

**Table 4:** Results of multilevel logistic regression analysis estimating the influence of key indicators on substance use at posttest

	Pretest use	Engagement		Class size
		Classroom	Individual	
	*AOR*	*AOR*	*AOR*	*OR*
Drink alcohol	4.71**	0.62*	0.60**	1.07
Get drunk	10.38**	0.67	0.53**	0.69
Smoke cigarettes	29.26**	0.60	0.33**	1.29
Use marijuana	34.79**	0.22+	0.14**	1.98+

Adjusted odd ratios (*AOR*) represent the change in likelihood of substance use at posttest associated with one standard deviation unit change in the independent variable. Classroom-level engagement and class size were standardized with respect to between-classroom variation

+*p* < .10. *p < .05. **p < .01

The zero-order correlation between *Antisocial Behavior* at pretest and individual student *Engagement* scores was − .18 (*p* < .01), suggesting that even before the program began, students who had lower levels of arguing, fighting, lying and cheating were more likely to be engaged in the intervention. Table 5 shows the results of the multilevel linear regression model predicting antisocial behavior. As depicted, both classroom- and individual-level engagement significantly predicted posttest antisocial behavior. Students from more engaged classrooms, as well as students who were personally more engaged relative to their peers, reported less antisocial behaviors at posttest. The sizes of these statistical associations were relatively small, however. The standardized effect sizes for both classroom- and individual-level engagement on antisocial behavior were smaller than the estimated effects of engagement on the mediators. Interestingly, for antisocial behaviors, classroom size played a marginal role in predicting the outcome.

**Table 5:** Results of multilevel linear regression estimating the influence of key indicators on antisocial behaviors

	Pretest	Engagement		Class Size	Variance explained	
		Classroom	Individual		Classroom	Individual
	Coef.	Coef.	Coef.	Coef.	%	%
Antisocial behavior	.43**	− .07*	− .22**	− .06+	86	24

*Coef*. = HLM-generated coefficient. All variables were standardized. Coefficients represent the standard deviation unit change in the dependent variable associated with a one standard deviation unit change in the independent variable. Classroom-level engagement and class size were standardized with respect to between-classroom variation. Variance explained was calculated by comparing the variance estimates for unconditional intercept-only models with estimates for models including pretest score, engagement at individual and classroom levels, and class size as predictors

+*p*<.10. *p < .05. **p < .01

## Discussion

In this study, we examined a simple hypothesis: students’ self-reports of engagement are related to proximal and distal outcomes in a drug prevention trial. This issue has rarely been examined empirically, given that most studies use teacher or observer ratings of student engagement, relying less on the students’ own thoughts and perceptions of the program’s features and teacher expertise. Quite naturally, students may differ in their motivation to learn and this has trickle down effects to whether they grasp the program content, enjoy the activities, and feel they benefit from participation. Overall, we found there is a significant relationship between students’ self-reports of engagement and program outcomes. The hypothesized active ingredients of the *All Stars Core* drug prevention program, including commitment, lifestyle incongruence, normative beliefs, and parental attentiveness were all statistically associated with students’ levels of engagement. We also observed strong empirical relationships between engagement and outcome measures, over and above that explained by pretest status and classroom size. Students who were in more engaged classrooms and those who reported more engagement relative to their peers reported higher posttest scores on the targeted intervening outcome measures.

There was also statistical evidence of the protective role of engagement in reducing antisocial behavior. As with the targeted intervening measures, there was a modest association between pretest status and engagement, the latter assessed at posttest. Nonetheless, the baseline adjusted regression coefficients corresponding to effects of classroom- and individual-level engagement on posttest antisocial behavior provide support for the protective role of student engagement in preventing or reducing the occurrence of antisocial behaviors. Even though these relations were statistically significant, they were relatively small in the current study.

There was less statistical support for the role of student engagement in the effectiveness of the intervention to reduce the onset of substance use. While effects were comparatively small, there was a discernable effect of classroom levels of engagement on alcohol use. Further, there were favorable program effects for engagement when modeled at the individual level on all substance use outcomes. Prevalence rates for substance use were quite nominal in the middle school population that participated in the *All Stars Core* trial. The low prevalence and lack of variability between classrooms limited our ability to draw firm statistical conclusions about the role of engagement on program behavioral outcomes. Studies that have a longer period of follow-up and that track youth through the critical developmental years when drug onset occurs would likely provide greater information regarding the importance of engagement in preventing substance use onset. At pretest, 5.3% of students reported drinking alcohol, 33.3% of whom quit at posttest. Of the students who had not had alcohol to drink at pretest, 4.7% reported doing so at posttest. At pretest, 1.6% of students reported getting drunk, of whom 20.0% had stopped at posttest. Among those not reporting getting drunk at pretest, 1.5% started doing so by posttest. At pretest, 0.9% reported smoking cigarettes, of whom 44.4% had quit at posttest. Among those who reported not smoking cigarettes at pretest, 1.6% initiated smoking by posttest.

### Classroom Versus Individual Effects

A reasonable question to ask is why the consistent results at the classroom level and the observation of variable findings at the individual student level? We posit that the enthusiasm and preparation of the teacher has a direct and notable effect on the class as a whole. These may reflect group norm effects, just as occurs in many social psychological studies of group influence (Ryan, 2001; Urberg, Değirmencioğlu, & Pilgrim, 1997). In the case of classroom instruction, some youth may be bolstered by the levels of classroom engagement, which helps them anchor their beliefs about drug use and the benefits of drug prevention. The classroom context also undergirds levels of trust between students and likewise between students and teachers. Students lacking trust in the school or not feeling connected with their educational milieu may shy away from engaging in drug prevention activities, despite the classroom engaging as a whole. Along these lines, one might expect that individual factors—temperament, academic ability, family background, etc.—would drive an individual’s response to a program. However, it may be that on occasion, individual factors simply fail to play a role and group norm effects driven by the teacher and coupled with a milieu of cooperative learning overshadow any individual effects.

### A Reliable Measure of Engagement

From a measurement standpoint, the use of posttest student survey items assessing engagement enhanced our ability to understand the degree to which students felt involved in the intervention. By virtue of the fact that this measure correlated with key outcomes, it appears superior to observational methods used in the past and that utilize expert judgments based on video-recorded instructional sessions (Bishop et al., 2014) and at least equal to observational methods employed by others (Pettigrew et al., 2015). The brief engagement scale was also easier to implement and, if incorporated into standard program evaluation surveys, can be employed for practically no additional cost. The proposed six-item measure of *Engagement* is reliable, valid at both the individual and classroom level and has utility. Importantly, the results provide evidence of construct validity for measuring engagement at both the individual and classroom level. This study is the first to report about these specific six engagement items. As a tool for evaluating intervention performance, this measure may prove valuable down the road for routine use in evaluating program delivery effectiveness.

### Student Engagement as Performance Feedback

The composite engagement scale may also offer trainers an opportunity to develop strategies for improving teacher performance. As a heuristic, we propose that values greater than 8 on the 0-to-10-point scale reflect a superior engagement outcome, which may serve as a useful benchmark to judge the adequacy of classroom engagement. (The value of 8 approximately equaled the value of 0 in Fig. 1, which was the value at which pretest-to-posttest improvement on proximal outcomes occurred when pretest proximal outcome scores were statistically partialed out.) We acknowledge that the value of 8 is arbitrary; however, if used to assess performance, teachers whose scores fall below this value may be encouraged to revisit their instructional methods when implementing a preventive intervention. At a minimum, a teacher’s scores would justify entering into additional training to promote increased engagement, primarily by encouraging the greater use of interactive teaching methods.

The items included in the engagement scale have a potential additional use. Each question in the scale might be useful for teachers to consider as performance feedback guides when they deliver prevention programs. Thus, teachers might improve their performance if they pay increased attention to students when they speak in class. For one thing, students notice when teachers are prepared to do a good job and they show this by paying attention, which contributes to mindfulness related to topics being presented. Adequate training and preparation may also help make the program enjoyable to students. *All Stars Core*, like many interactive prevention programs, encourages students to enter into discussions and share their opinions. Creating an overall quality experience that encourages students to pay attention is a key factor to increasing engagement. Finally, teachers may wish to help students focus on long-term program goals. In the case of *All Stars Core*, this included helping students think about what was important to them as they grew older. Training in prevention often focuses on introducing teachers to new and different and more interactive mechanics of instruction. This is above and beyond their standard academic discipline curriculum-focused training that emphasizes less interaction and more didactic expression. In this respect, finding ways to expand teacher training to include an increased awareness and understanding of the importance of developing student engagement is certainly warranted.

### Does Classroom Size Matter?

The findings regarding the influence of classroom size were of secondary importance in this project, included primarily as a plausible rival explanation to engagement. Classroom size was not a meaningful correlate of most proximal and distal outcomes. With the exception of the antisocial behavior analysis, the relation between engagement and classroom size became somewhat trivial once we modeled pretest values and targeted program outcomes. In the case of antisocial behaviors, smaller classes generally performed less well than average sized classes. We speculate that the poor effects observed with relatively small classes may have something to do with classroom composition. It is possible, for example, that there might be a selection bias working in the creation of ultra-small classes if these classes are composed primarily of higher-risk individuals with academic or behavioral deficiencies (Dishion & Dodge, 2005). It is also possible that *All Stars Core* simply works less efficiently when there are too few students in a classroom and both students and teachers struggle with class dynamics. The program includes a number of small group activities that work best when sufficient numbers of students are present. For example, having a suitable number of students helps ensure that discussions designed to reveal class norms carry sufficient weight for students to understand intended messages. Too few students may alter this dynamic to the point where small classes don’t benefit from this portion of the *All Stars Core* curriculum. It should be noted that classroom sizes in this project did not approach what previous studies considered large, which is typically over 30 students. Thus, the relative restriction on range does not allow us to comment on this issue generally. In particular, we were unable to estimate how very large classrooms would respond to *All Stars Core*.

### Study Limitations

There are a number of study limitations worth noting. No matter which strategy or conceptual framework is used to posit measures of student engagement, it is a complex multidimensional construct (Appleton, Christenson, Kim, & Reschly, 2006). We used a very brief assessment capturing at most one or two facets of student engagement, leaving open the door that other components of engagement may also play a role in program outcomes. Moreover, some have argued that researchers have neither adequately defined engagement nor demonstrated its divergent validity from motivation, competence, or autonomy, and even from school bonding or attachment (Appleton et al., 2008; Furlong et al., 2003). In theory, students who persist on tasks, find the problem interesting if not challenging, can work autonomously, and feel a sense of relatedness between their school work and their personal life are more likely to be highly engaged. This is in a large part the crux of self-determination theory (Deci & Ryan, 1985; Ryan & Deci, 2000), which posits that some students are intrinsically motivated for success. In this respect, a lengthier assessment that includes measurement of these different cognitive, affective, and behavioral facets of engagement may increase the model’s predictive validity. We also neglected the social context of schools as a possible source of engagement. Here again, expanded definitions and conceptual models for student engagement attribute some portion of academic success to environmental factors like the class composition and social environment (Ryan & Patrick, 2001) as well as teacher support and guidance (Gregory & Ripski, 2008; Wentzel, 1997), all of which influence health risk behaviors (Griffiths et al., 2012) and educational outcomes (Dotterer & Lowe, 2011; Furlong et al., 2003).

We also relied on students’ reported levels of engagement introducing a modicum of single-source method variance into the model. Parents and teachers can provide additional information that yields a more complete picture of a student’s levels of engagement. Teachers know when students are bored, drift mentally or lose interest in classroom activities, whether they are completing class assignments, finishing homework, attending to classroom instruction, and whether the student is attentive or disruptive. They can also monitor day-to-day social interactions among students through classroom interactions that boost their understanding of cliques and social networks that form in schools. Likewise, parents have an implicit sense of their child’s academic proficiency, time spent on task, and other relevant features that are indicative of the level of attachment to the school and educational goals (e.g., participating in extracurricular activities). Many of these signals are formative pieces that help round out our understanding of engagement (Griffiths et al., 2012) and are readily available to independent observers. Having these independent reports based on observable behaviors would provide a more robust sense of engagement.

Students self-reported their substance use involvement. Because overall rates of substance use were low, it is possible that students under-reported their use. Early research (Evans, Hansen, & Mittelmark, 1977; Murray, O’Connell, Schmid, & Perry, 1987) suggested that there may be a tendency by youth to under-report. Other researchers examining validity of adolescent self-report have not found significant bias (Hansen, Malotte, & Fielding, 1985; O’Malley, Bachman, & Johnston, 1983; Winters, Stinchfield, Henly, & Schwartz, 1990). With no corollary biological tests completed, we cannot know for certainty if under-reporting occurred. We do believe, however, that there was no over-reporting.

Admittedly, we may have only brushed the surface of the complex mechanisms underlying student engagement. For instance, we did not posit any temporal relations between engagement and both proximal and distal outcomes as we waited until after the program was administered to assess the students’ perspectives on the quality of the delivery of the program and their overall satisfaction. The educational literature espouses a model of school connectedness tied to academic outcomes suggesting that school connectedness reflects three components: support, belonging, and engagement (Appleton et al., 2008; Connell & Wellborn, 1991). The causal sequence involves students feeling empathy from their teacher, receiving attention and praise from their teacher, which fuels a sense of belonging to the school community or classroom (i.e., identification and participation). In turn students increase their engagement and academic motivation to do well, thus improving their performance (Dotterer & Lowe, 2011; McNeely & Falci, 2004). Only with additional data that allow temporal spacing between the engagement measure, postulated causal mediators, and drug program outcomes can we test a model positing domino effects of this nature.

We also used a modified form of threshold analysis in determining the minimal recommended cut-point for levels of student engagement. This is a very subjective cut-point as is often encountered in a regression discontinuity design. Shifting the threshold a modest amount to the left or right can either lead to a gain or reduction in model precision. Either way, the empirical threshold is sample-specific and makes it hard for others to replicate the same findings. As a result, and given the analysis strategy taken, we know that student engagement affects program outcomes in a meaningful way, but we do not know what precise level of engagement matters.

## Conclusion

This study addresses the psychometric and statistical utility of using a self-report method for assessing student engagement in an evidence-based and widely disseminated drug prevention program, *All Stars Core*. The measure of student engagement was statistically reliable. Higher levels of engagement were associated with posttest improvements in targeted risk factors and antisocial behaviors and, to a lesser degree, with reduced onset of substance use. There were, however, noted differences in whether engagement was assessed at the classroom versus the student level, suggesting that classroom dynamics also play an integral role in the success of prevention programs. The findings suggest that actively engaging students in prevention should be included in teacher training and local program evaluation. Strategies for improving engagement should be developed and tested. Prevention programs may also want to include program material that addresses cooperative learning instructional strategies to increase classroom (if not school) social cohesion and improve learning outcomes. Even faced with the current findings, a theoretical framework that links the different ways engagement works with program efficacy needs to be further developed so that we can ensure that programs going to scale are guided by a unified conceptual model.

Requested Additional Statements.

1. Research involving human participants and/or animals: This paper involves data provided by adolescents. Data were collected from students as part of a project being conducted by Barnardo’s Northern Ireland. Data were collected in 62 classrooms in which *All Stars Core* (Hansen, 2015) was delivered. Data were available from 980 students.
2. Disclosure of potential conflicts of interest: Between 2010 and 2015, Author A (Hansen) had contracts from Barnardo’s Northern Ireland to purchase *All Stars Core* materials, train teachers and design and print student surveys. Barnardo’s is the sole owner of data used in this project and its use was granted free of charge for the purposes of completing the analyses presented in this research article. *All Stars Core* was developed by Author A (Hansen), but there is currently no financial relationship between him and the company that markets and distributes *All Stars Core*.

## Appendix

### Proximal Variable Survey Prompts

Except for normative beliefs about substance use items 2, 3, and 5 and normative beliefs about fighting item 4, responses categories included “Strongly disagree,” “Disagree,” “Agree,” and “Strongly agree” with values for each item of 1, 3.33, 6,67, and 10, respectively except when reversed. Normative beliefs items used the following response categories: “None,” “Some,” “Half,” “Most,” and “All” with values of 10, 7.5, 5, 2.5, and 0, respectively.

#### Commitment to Avoid Substance Use

1. I have made a final decision to stay away from cannabis (or dope).
2. I will smoke cigarettes sometime in the future (reversed).
3. If I had the chance and knew I would not be caught, I would drink an alcoholic beverage (reversed).
4. I would be willing to sign my name to a pledge saying that I will never use alcohol.
5. I have made a decision not to get high by sniffing fumes.
6. I have made a promise to myself that I will never drink alcohol at all OR that I will wait until I am 18 years old.
7. I have told at least one person that I do not plan to smoke.
8. It is clear to my friends that I am committed to living a drug-free life.

#### Commitment to Avoid Fighting

1. I have made a strong decision to walk away rather than fight.
2. I am committed to settling arguments without physically hurting others.
3. I have decided that I will never physically hurt someone else on purpose.
4. If someone does something that bothers me, I will fight them (reversed).

#### Lifestyle Incongruence with Substance Use

1. I will have a happier life if I stay away from alcohol.
2. Using cannabis (or dope) would keep me from accomplishing my goals.
3. If I started using cannabis (or dope) at my age, it would harm the quality of my life.
4. Smoking cigarettes fits with the kind of life I would like to live (reversed).
5. Getting drunk every now and then fits with the kind of life I want to lead in the future (reversed).
6. Getting high from sniffing glue would get in the way of what is important to me.

#### Lifestyle Incongruence with Fighting

1. Getting into fights fits with the kind of life I want to have (reversed).
2. To live the life I want, I must always solve problems peacefully and not fight.

#### Normative Beliefs About Substance Use

1. My friends think sniffing glue or fumes to get high is really fun (reversed).
2. How many people your age do you think get drunk at least once a month?
3. How many people your age do you think smoke cigarettes regularly?
4. My friends think smoking cannabis (or dope) is a stupid thing to do.
5. How many people your age do you think use cannabis (or dope) at least once a month?
6. My friends think smoking cigarettes is cool (reversed).
7. My friends think it is OK to get drunk every now and then (reversed).

#### Normative Beliefs About Fighting

1. My friends think fighting is an OK way to settle differences (reversed).
2. Most people my age stay away from getting into fights.
3. My friends think people who pick fights are really stupid.
4. How many people your age do you think have been in a fight where people hit each other in the past month.

#### Parental Attentiveness

1. I have talked with my parents about living a drug-free life.
2. My parents often talk with me about what they think is important.
3. My parents expect me to NOT drink alcoholic beverages as a teenager.
4. My parents and I talk together about things that are important to me.
5. My parents always know who I’m with.
6. My parents know who my friends are.
7. My parents always know what I`m doing when I`m with my friends.

### Engagement Prompts, Response Categories and Weights (in Parentheses)

Did your teacher pay attention to you when you spoke in class?

1. No (0)
2. Yes, a little (5)
3. Yes, a lot (10)

Did your teacher do a good job teaching All Stars?

1. Definitely yes (10)
2. Mostly yes (6.67)
3. Mostly no (3.33)
4. Definitely no (0)

Did you enjoy All Stars?

1. No (0)
2. Yes, a little (5)
3. Yes, a lot (10)

How often did you share your opinion during All Stars?

1. Never (0)
2. Rarely (3.33)
3. Occasionally (6.67)
4. Often (10)

How often did you pay attention during All Stars?

1. Never (0)
2. Rarely (3.33)
3. Occasionally (6.67)
4. Often (10)

Did All Stars help you think about what was important to you as you grow older?

1. No (0)
2. Yes, a little (5)
3. Yes, a lot (10)

## Footnotes

1 We use the term “authoritative” in a manner consistent with studies of parenting and child development, which discern between authoritarian and authoritative parents (Baumrind, 1966, 1971). The authoritative teacher is fair, democratic, reasons with the student, encourages self-reliance and individuality, is responsive to the students’ needs but is also firm and has high standards.
